# Research on the influence of relocation adaptability on the employment stability of Chinese-style labor immigration: The case of labor migrant communities in Yinchuan City, Ningxia, China

**DOI:** 10.1371/journal.pone.0304199

**Published:** 2024-06-06

**Authors:** Jianrong Fan, Wanyu Du, Zhennan Fan

**Affiliations:** 1 School of Economics, North Minzu University, Yinchuan, China; 2 College of Economics and Management, China Three Gorges University, Yichang, China; Caleb University, NIGERIA

## Abstract

Achieving full and stable employment is not only one of the goals of macro-control by governments but also a key issue that labor migrants must address. To understand the impact of relocation adaptation on the employment stability of Chinese-style labor migrants, members of the group visited the labor migrant settlement sites in Yinchuan City and used questionnaires to investigate the adaptation and employment status of farmers after relocation. The article attempts to analyze the impact of relocation adaptability, embodied by social adaptability, economic adaptability, and cultural adaptability, on the employment stability of Chinese-style labor migrants using structural equation modeling with the highly representative field research data from the labor migrant community in Yinchuan City as an example. The results of the study show that the social, economic, and cultural adaptability dimensions of relocation adaptability all have a significant positive effect on employment stability. Therefore, to promote the stable employment of Chinese-style labor migrants, it is necessary to enhance economic adaptability to stabilize employment and increase income, enhance social adaptability to proactively adapt and actively participate, and strengthen cultural adaptability to proactively seek change and actively adapt to better improve the employment situation of labor migrants in an orderly manner.

## Introduction

Stabilizing employment is not only one of the macroeconomic control objectives of the global economy—full employment—but also an important way to effectively eradicate poverty among vulnerable groups. Since 1978, when the United Nations Conference on Environment and Development first formally proposed the concept of sustainable development to the international community, all countries have been exploring and practicing this approach, and a consensus was formed at the 1992 United Nations Conference on Environment and Development and the 1994 United Nations Conference on Population and Development, both of which particularly emphasized that "the center of sustainable development is the human being" as well as the "people-centered concept of sustainable development." Combating poverty has been a key issue in the governance of nations in both ancient and modern times, and China has always been an active advocate and a powerful promoter of the cause of poverty reduction around the world. In 2003, China also proposed a scientific concept of development, the core content of which is “people-orientedness” and “comprehensive, coordinated, and sustainable development.” China is the world’s largest developing country, and the implementation of relocation for poverty alleviation is a typical example of adherence to people-centered, sustainable development and a fundamental way to solve the problem of one side of the country being unable to feed its people well and to realize leapfrog development for the impoverished masses.

The academic definition of "labor migration" can be broadly categorized into three types. The first is the internationally recognized skilled migration, including domestic and cross-border skilled labor migration. The second refers to the group of people who migrate from rural communities to urban communities in China to seek work and increase their income, whom some scholars refer to as "migrant workers." These first two types are forms of economic migration undertaken by labor migrants themselves in accordance with the trend of marketization and their own preferences. The third category refers specifically to planned and organized migration by the government. This is the category to which this study belongs.

Specifically, Chinese-style labor migration refers to the following: to effectively solve the problem of absolute poverty in the region, through administrative means and policy support and on the premise of providing a certain degree of employment security, housing security, social security, etc., the relevant government departments will, in a planned and systematic manner, relocate poor people in areas that are unsuitable for human production and survival to the urban communities in the region where the environment is favorable and where there are more opportunities for development to rapidly realize poverty eradication. This group of migrants is formed to realize sustainable development.

Reflecting on the history of poverty alleviation and development in China, the "Hangzhuang Migration" poverty alleviation project, which began in 1983 in Ningxia, Gansu, and other "Three Wests" areas, was a precedent for relocating people to alleviate poverty. From 2001 to 2015, the National Development and Reform Commission (NDRC) organized and implemented the relocation project for poverty alleviation, relocating more than 6.8 million people in poverty-stricken areas over a period of 15 years. From 2015 to 2020, relocation for poverty alleviation became one of the Chinese Government’s key means of effectively addressing the problem of absolute poverty, with a cumulative investment of RMB 600 billion, the relocation of 10 million poor people, and the formation of 35,000 communities for poverty alleviation, including 5,000 urban communities.

The Ningxia Hui Autonomous Region (hereinafter referred to as "Ningxia") is located in the northwest of China. It is not only a key province and region of contiguous poverty alleviation and development in China, but also one of the pioneering areas of poverty alleviation and relocation in inhospitable areas in China. In particular, the problem of absolute poverty in the southern region of Xihaigu (hereinafter referred to as "Xihaigu") is extremely serious. Xihaigu is the collective name of Xiji, Haiyuan, Guyuan Yuanzhou District, Pengyang, Tongxin, Jingyuan County, Lund County, and other poor counties at the national level in the loess hilly area, and in 1972, the United Nations Food and Development Organization (FDA) identified it as an "area unfit for human survival." "Xihaigu" has become nearly synonymous with "poverty." The natural conditions there are harsh, with prolonged drought, serious soil erosion, average annual precipitation of about 300 millimeters but evaporation of more than 1,000 millimeters, and a variety of other natural disasters. These areas are predominantly mountainous, limited by geography, with backward infrastructure, weak production capacity, and poor education and medical conditions, and the task of poverty alleviation is arduous. The implementation of the relocation project for poverty alleviation and helping the poor population in areas with poor living conditions to eradicate poverty is the shortest of the shortcomings to be remedied.

To effectively solve this problem, the Party Committee and government of the Ningxia Autonomous Region, under the guidance of the State Council’s "Three West" Office’s guideline of "taking the water route if there is a water route, taking the dry route if there is a dry route, and looking for other routes if there is a difference between the water and the drought," initiated the prelude to the region’s poverty alleviation and relocation activities: offsite poverty alleviation and development in 1983, which has not only enabled most of the relocated poor households to realize their wish to escape from poverty and become rich and has achieved favorable social, economic, and ecological benefits but also has led to the experience of Ningxia rising to the level of a national strategy at the beginning of the current century—the strategy of relocation to alleviate poverty—which has become an important measure for the implementation of precision poverty alleviation and precise poverty alleviation in the period of the Thirteen-Five-Year Plan.

In the face of an increasingly severe migration situation, Ningxia has begun to explore a new "land for ecology, land for labor" mode of relocation for poverty alleviation: the labor migration mode. So-called "labor migration" at all levels of government assistance will not be suitable for the survival and development of the ecological environment, which is extremely poor. The poor people in the southern mountainous areas of Ningxia organized relocation for the development of a relatively good environment that is suitable for the development of the northern part of Ningxia Chuan District towns and their surrounding areas as well as to provide a certain amount of housing and certain employment opportunities. To this end, they engaged in the development of non-agricultural industries, employment opportunities, and non-agricultural industry development to help realize the goal of poverty alleviation migration. It is quite different from the internationally accepted high-skilled labor migration, that is, the Ningxia labor migration studied in this paper belongs to the category of low-skilled migration.

Although labor migration is a typical governmental act, labor migrants, as the main actors of relocation, must gradually adapt to this change. On the one hand, under the guidance and support of government policies, labor migrants need to actively adapt to the evolving situation, change the poor and backward situation through relocation, and integrate into the social development of the place of relocation as soon as possible to seek better development opportunities. On the other hand, labor migrants are faced with a relatively unfamiliar urban development environment and need to adapt passively to the predicament, especially in the absence of effective guidance, and this passive state may persist longer.

The "landless resettlement" of labor migration is an innovation in the mode of relocation for poverty alleviation, and although initial results have been obtained, it is new, is subject to the influence of the macroeconomic situation and personal factors, and faces severe tests, the most prominent being the problem of employment and employment stability. The ability to achieve stable employment and improve employment stability has become the key to the sustainable development of labor migration.

Located in the middle reaches of the Yellow River in Ningxia, Yinchuan is relatively rich in water and land resources, which provides a good foundation for poverty alleviation relocation. As the political, economic and cultural center of Ningxia, Yinchuan not only has a more complete system of policy support and service guarantee to better accommodate and resettle the relocated households but also has a more complete industrial system and market environment to provide abundant employment opportunities and has complete facilities in education, healthcare, and other social services, providing good public service resources for the relocated households. As a result, it has been one of the most important resettlement sites for the Ningxia government to implement relocation for poverty alleviation, and several labor migration communities have been formed to provide a better living environment and development opportunities for relocated households from poor areas, helping them escape from poverty and become rich.

Based on the above reasons, to do a good job of researching the adaptability and employment stability of labor migrants in resettlement sites, this study selects a relatively large-scale labor migrant community in Yinchuan City, Ningxia, with which is easy to carry out questionnaire surveys, as the research object to evaluate the three dimensions of the economic, cultural, and social adaptability of labor migrants. Economic adaptability includes income mode, consumption mode, and employment mode; cultural adaptability includes language and relationships with local people; and social adaptability includes ecological environment, climatic conditions, and management mode. Through the analysis of the evaluation results, the problems and causes of labor migrants’ adaptability can be revealed, and then corresponding measures can be taken to improve the relocation adaptability of labor migrants, promote their stable employment, narrow the gap between the rich and the poor among different social groups, and achieve the goal of common prosperity.

## Literature and hypotheses

### 2.1 Literature review

Migration is an effective way to achieve the utilization of human resources and improve the reallocation of resources [1].The employment of labor migrants has been widely addressed, and the research on employment stability mainly includes the measurement, trends and factors affecting this issue. The academic evaluation indicators of employment stability are often diverse, and the representative indicators include the job tenure of workers, current job retention rate [2], the proportion of temporary dismissals [3], and the number of work stoppers [4], length of the tenure and working hours [5], years of employment and separation rate [6], employment change rate, employment elasticity coefficient, ratio of final employment rate to base period employment rate, change rate of employment time [7], separation rate [8], promotion [9]. As for the trend of employment stability, most studies showed that employment stability showed a downward trend. Polsky [10], Neumark [11], Farber [12]and other scholars found that job stability in the American labor market showed a gradual downward trend. Burgess and Rees [13], Marcotte [14]; Gregg and Wadsworth [15] and other scholars also found the same trend in the study of employment stability in the UK. The study of employment stability in France by Givor and Maurin [16] also showed that the risk of involuntary dimssion in France in the 1990s increased compared with that in the 1980s. Annette Bergemann and Antje Mertens [17] studied the German labor market and found that in the 1980s and 1990s, the job stability of male employees between the ages of 16 and 56 showed a downward trend. Kato [18]compared the stability of employment in Japan before and after 1987 and found that after the bursting of the economic bubble, the long-term employment system began to break down in Japan, and the job instability of young employees increased, but the job stability of old employees, the core labor force of enterprises, did not change significantly. Joonmo Cho and Jaeho Keum [19] used Korea’s panel data on labor and income to measure the dynamic changes of employment stability in the labor market between the 1997 financial crisis and the recovery. And it never returned to its original level. Scholars also analyzed the factors affecting employment stability. Muchinsky et al [20] believed that working relationship, economic situation and personal characteristics were three factors affecting employment stability. Deshpande [21] believed that job satisfaction was negatively correlated with turnover intention. Price, J.L. [22]and Diprete, T.A. [23]found that the continuous updating of social technology will directly lead to the decline of employment stability. Ljungqvist, L. [24]believed that the decline in employment stability was mainly driven by the employment flexibility policy. Forslund et al [25]concluded that the stability of employment positions and labor relations were the basic needs of workers in employment. E.Jacobs and G.Roodt [26] Georgia Pomaki et al. [27] Robert J. Blomme et al. [28] From the aspects of organizational culture and environment, it is found that employees’ dimission intention is related to the degree of fit between individuals and organizations, and is correlated with organizational culture, environment, individual characteristics and other factors in a large or small, positive or reverse way. Fu, Yuming, and Gabriel (2012) argued that differences in the regional distribution of human capital are associated with migrants’ employment choices and regional economic differences [29].

Relocation adaptability is of great significance to labor migrants, who can successfully adapt themselves to the changing situation through the control of language, customs, laws and social systems. Social adaptability is an important part of psychological and sociological research. The word "adaptation" was first proposed by Herbert Spencer and applied to the social field. He believed that social adaptation refers to a state in which human beings can continuously adjust themselves according to the changes of the external living environment and finally achieve a harmonious coexistence with the new living environment, scholars are based on this definition to conduct analysis. Zhao and Yaohui noted that the main barriers to migration are a lack of safety during transportation and in the destination city and forced separation from family members [30].Carl F believed that adaptation is a kind of ability, which needs to be further improved by changing one’s own ability [31]. Erisken believed that adaptation is bidirectional, and it is necessary to clarify the content and direction of adaptation [32]. As for the research content of social adaptability, Meyers et al., an early researcher of social adaptation, believed that adaptive behavior consists of two basic components: self-satisfaction and social responsibility. Scholars have concreted it into four aspects: politics, economy, culture and environment. Especially in the research of social adaptability factors, domestic scholars combined the big Five personality theory of Buss (1989), an American psychologist, with China’s unique cultural characteristics and proposed that social adaptability be explained as five factors, including social interaction, social tolerance, responsibility, stability and openness to exploration. These five aspects covered the main content of a good social adaptability personality.

In summary, based on the previous research results and the practice of labor immigrants in Ningxia, this study shows that the employment stability of labor immigrants examined here is the self-evaluation of individual labor immigrants who can obtain relatively stable income through employment in a certain period of time, maintain family life at a certain level, and transition between employment and non-employment. It is manifested as a type of intentionality and tendency. Based on the actual employment situation of labor immigrants, this study constructs an index system to measure employment stability based on relocation adaptability, which is reflected by social, economic, and cultural adaptability, and four observational variables, namely self-evaluation of the employment stability of labor immigrants, employment policy satisfaction, employment mode adaptability, and family members’ employment satisfaction.

### 2.2 Hypotheses analysis

#### 2.2.1 The relationship between economic adaptability and employment stability

Economic adaptability refers to the ability of individuals to obtain or dispose of income through their own efforts in the process of adapting to the development environment of the region in which they live. For labor migrants, economic adaptability mainly includes adaptability of employment, of income sources, and of consumption patterns. First, labor migrants need to adapt to changes in employment mode, revise the previous employment concept of "born to be a farmer with a job" and "waiting for a job to come to the door," and vigorously realize active employment through various approaches. Second, labor migrants also need to adapt to changes in the source of income; they must no longer rely on the mode of "selling land for income" but actively participate in employment activities to obtain due labor compensation. Third, labor migrants need to adapt to changes in the way they consume, shifting from the old "rural consumption pattern based on the output of the land" to the more modern "urban consumption pattern based on monetary expenditures" on the premise of obtaining remuneration through active employment. Only by constantly adapting to new changes in the development environment, upgrading their abilities, and adapting to social and economic changes can labor migrants respond flexibly to new opportunities and challenges and achieve sustainable economic development.

Economic adaptability and employment stability are closely linked. Economic adaptability refers to the ability of an economy to adapt to changes in the external environment, while employment stability refers to the ability of an economy to maintain employment levels. The relationship between the two is interdependent, as higher economic adaptability can help an economy cope with changes in the external environment and thus increase the level of employment. Conversely, higher employment stability can also help an economy better adapt to changes in the external environment, thus increasing economic adaptability. Accordingly, the following hypotheses are formulated:

**H1.** *An increase in economic adaptability leads to an increase in employment stability*.

#### 2.2.2 The relationship between cultural adaptability and employment stability

Cultural adaptability refers to the ability of an individual’s cultural situation to adapt to the environment and harmonize with other cultures. For labor migrants, cultural adaptability mainly includes adaptability to the language environment, adaptability to the relationship with local people, and adaptability to the way of interaction.First,continuously improving the ability to adapt to the language environment is the fundamental place for labor migrants to strengthen communication and establish a foothold in development. The language of the labor migrants in Ningxia is the same with that of the destination, so the development of labor migrants is more ideal.Second,continuously enhancing the relationship with the locals is an important prerequisite for labor migrants to expand their horizons and enhance cooperation, as the saying goes, "one more friend, one more road", there will be more options to strengthen the connection with the local people.Third, constantly adapting to the diversity of ways of communication is an important basis for labor migrants to meet needs and seek opportunities. There are significant differences in communication approaches between people, and constantly adjusting and adapting provides more opportunities for development. Labor migrants can make more friends and create more employment opportunities only if they take the initiative to adapt to changes in their new cultural environment and increase their own cultural construction and adaptation so as to enhance the stability of their employment.

A close link exists between cultural adaptation and employment stability. Cultural adaptability refers to a person’s ability to adapt to different cultural contexts, while employment stability refers to a person’s ability to maintain employment in a particular cultural environment. Therefore, cultural adaptability can help a person better adapt to different cultural environments and thus increase employment stability. Accordingly, the following hypothesis is proposed:

**H2.** *An increase in cultural adaptability leads to an increase in employment stability*.

#### 2.2.3 The relationship between social adaptability and employment stability

Social adaptability refers to an individual’s ability to harmonize and adapt to the social environment, including interpersonal communication, social adaptation, and other aspects. For labor migrants, social adaptability mainly includes the adaptability of immigrant status, the adaptability to being discriminated against by others, and the adaptability of spare time in life. First, we the special characteristics of immigrant status should be diluted, and local residents should actively participate in social activities; second, the dilemma of being discriminated against by others should be lessened, and the unfavorable situation brought about by misunderstanding should be positively resolved with a normal mindset; third, the homogeneous nature of spare time should be strengthened, and individuals should integrate into colorful social activities as a local citizen. Only by actively adapting to changes in the new social environment and increasing mutual respect and harmonious coexistence with others can labor migrants seek more employment opportunities and enhance the stability of their employment.

There is a close link between social adaptability and employment stability. Social adaptability refers to an individual’s ability to adapt to the social environment, while employment stability refers to an individual’s ability to maintain employment in society. The stronger the social adaptability is, the more likely the individual will be able to obtain stable employment opportunities, thus increasing employment stability. In addition, social adaptability can also help individuals better adapt to the social environment, thus achieving employment stability. Accordingly, the following hypothesis is proposed:

**H3.** *An increase in social adaptability leads to an increase in employment stability*.

#### 2.2.4 The three dimensions of relocation adaptability synergistically affect the employment stability of labor migrants

A close relationship exists among the three dimensions of relocation adaptability, namely, "economic adaptability," "cultural adaptability," and "social adaptability," which are mutually supportive and mutually reinforcing. Economic adaptability refers to the ability of a society or organization to adapt to economic changes, and the stronger this ability is, the more it can provide a strong economic foundation for cultural and social adaptability. Cultural adaptability refers to the ability of a society or organization to adapt to cultural changes, and again, the stronger this ability is, the more it can provide a good cultural foundation for economic and social adaptability. Finally, social adaptability refers to the ability of a society or organization to adapt to social changes, and the stronger this ability is, the more it can provide a good cultural foundation for economic adaptability and social adaptability. Social adaptability refers to the ability of a society or organization to adapt to social changes, and the stronger this ability is, the more it can lay a solid social foundation for economic adaptability and cultural adaptation. The relationship among these three is interrelated can be summarized in one sentence: economic adaptation, cultural adaptation, and social adaptation are three important aspects of social development, and together, they contribute to the improvement of the level of stability in the employment of labor migrants.

Structural equation modeling (SEM), also known as covariance structure analysis, is a multivariate statistical method and has become increasingly mature after years of practice and development. It is widely used in economics, management, psychology, education, and other fields and especially in satisfaction research. The research results are reasonable and reliable, can handle measurement and analysis problems at the same time, and have theoretical apriority. SEM allows measurement errors between independent variables and dependent variables and analyzes the relationships among potential variables by measuring observable variables.

**Fig 1:**
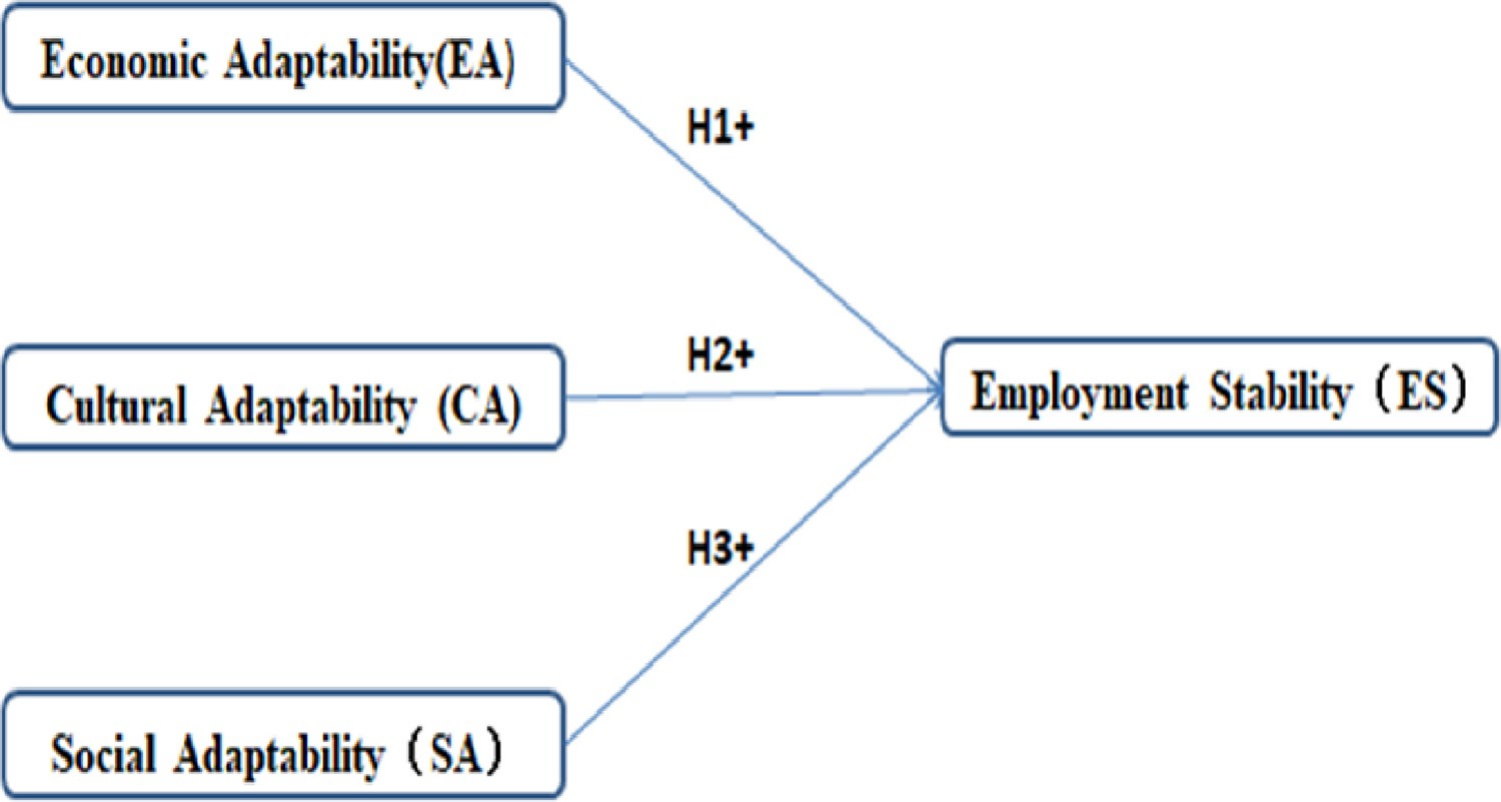
Structural equation model diagram.

Based on the principle of SEM, this paper constructs the initial structural equation model of relocation adaptability and employment stability, as shown in Fig 1.

## Design

### 3.1 Scale and questionnaire design

The scales used in this study are based on the existing relevant literature, combined with the specific theory and practice of labor migration in Ningxia, and adjusted and modified according to the actual situation of the surveyed area and the needs of the research. The questionnaire is divided into three parts. The first part is the personal and family characteristics of the survey object, including the gender, age, education level, and basic economic status of the family. The second part covers relocation adaptability, including economic adaptability, social adaptability, and cultural adaptability. The third part focuses on employment stability, including employment self-evaluation, employment satisfaction of family members, and employment environment satisfaction. All constructs are measured on a five-point Likert scale, and the possible answers are “Very satisfied,” “Satisfied,” “Neutral,” “Dissatisfied,” and “Very dissatisfied,” with corresponding scores of 5, 4, 3, 2, and 1. Each latent variable and its observed variable indicators are shown in Table 1.

**Table 1:** Summary of research variables and observed variables.

Latent Variable			Observed Variable
Relocation adaptability (RA)	Economic adaptability (EA)	EA1	My comfort level with my existing income is
		EA2	My adaptability to the existing employment mode is
		EA3	My adaptability to existing consumption patterns is
	Culture adaptability (CA)	CA1	My adaptability to existing part-time life is
		CA2	My adaptability to being discriminated against is
		CA3	My adaptability to existing immigration status is
	Social adaptability (SA)	SA1	My adaptability to existing and local people is
		SA2	My adaptability to the existing language environment is
		SA3	My adaptability to the way I interact is
Employment stability (ES)	Employment stability (ES)	ES1	My satisfaction with the existing employment environment is
		ES2	My family members’ employment satisfaction status is
		ES3	My satisfaction with the existing employment policy is
		ES4	My assessment of my employment stability is

### 3.2 Data collection

Yinchuan City, as the capital of the autonomous region, is also the most economically and socially developed region in Ningxia, with better economic development conditions and a higher level of urbanization, and thus has always been one of the key areas for ecological and labor migration resettlement in Ningxia. There are labor migration resettlement sites in three districts (Xingqing, Jinfeng, and Xixia), two counties (Helan and Yongning) and one city (Lingwu) under the jurisdiction of Yinchuan City. By the end of 2020, there were 18 labor migration communities in Yinchuan City, resettling 7,284 households and 28,732 people. They accounted for nearly one third of the total number of labor migrants in the whole region of Ningxia, and their number ranked first in that area.

The formal research was conducted in mid- to late-September 2020. Based on the experience of the pre-survey, the impact of the epidemic, the convenience of the research, and the characteristics of the work of labor migrants (the research can only be conducted from 19:00 to 21:00), one-on-one household questionnaire surveys were carried out through Questionnaire Star for the sample villages with the cooperation of the Poverty Relief Office of Yinchuan City in Ningxia, which is now renamed the Bureau of Rural Revitalization and the grass-roots community organizations. A total of 415 questionnaires were returned, with a recovery rate of 100%. The number of qualified questionnaires after statistical screening was 387, and the validity rate of the questionnaires was 93.25%. The questionnaire consists of two parts, the basic information of migrants and the main questionnaire, which covers three aspects: social adaptation, employment, and family characteristics.

Table 2 shows the basic information of immigrants. It shows that among the respondents, the gender ratio is basically the same, with males accounting for 50.39% and females for 49.61%. In terms of age, 58.14% were 30–45 years old, 17.31% were younger than 29 years old, and 24.55% were older than 46 years old. Regarding marital status, 90.18% were married, which meets the basic requirements of labor migration. The unmarried people were likely to be adult children of labor migration, accounting for 9.82%; 75.45% of those who have been migrating for less than 4 years, and 24.95 of those who have been migrating for more than 5 years, which is consistent with the migration period of Ningxia and the regional distribution of respondents mentioned above. Among them, Minning Town was the immigrant during the 12th Five-Year Plan period, while Zhangzheng Town and Wangyuan Town were the immigrant during the 13th Five-Year Plan period. In the ethnic structure, the Han nationality accounts for 43.93%, and the Hui nationality accounts for 56.07%. In general, this survey includes migrants of different genders, ages, nationalities, and relocation periods, which is highly random and representative.

**Table 2:** Sample demographic data. (n = 387).

Characteristic	Range	Frequency (person)	Percentage (%)
Gender	Female	192	49.61
	Male	195	50.39
Age	<29	67	17.31
	30–45	225	58.14
	>46	95	24.55
Marriage	Unmarried	38	9.82
	Married	349	90.18
Time of migration	<4	292	75.45
	>5	95	24.55
Ethnic group	Hui nationality	217	56.07
	Han nationality	170	43.93

## Results

### 4.1 Structural equation model

Labor migrants are a special vulnerable group; the evaluation of their social adaptability is highly uncertain and complex, and there are problems that are difficult to quantify. It is difficult to accurately estimate the relationship among latent variables with ordinary regression models, while structural equation models integrate regression analysis, factor analysis, and path analysis. The correctness of the model and the reasonableness of the model hypothesis can be verified according to the internal structural relationship among the variables. It consists of two parts: a measurement model and a structure model. The measurement model represents the relationship between latent variables and observed variables. Structural equation models can reflect the relationship between potential variables. The specific model expression is as follows:

Χ=Λxξ+δ (1)

Y=Λyη+ε (2)

η=Βη+Γξ+ζ (3)

Eqs (1) and (2) are measurement models, where *X* is the observable variable of the exogenous latent variable, *Y* is the observable variable of the endogenous latent variable, *η* is the endogenous latent variable, *ξ* is the exogenous latent variable, *Λx* is the association coefficient matrix of the exogenous latent variable and the observable variable, *Λy* is the association coefficient matrix of the endogenous latent variable and the observable variable, and *δ* and ε are error vectors. Eq (3) is a structural model reflecting the interaction between exogenous and endogenous latent variables where *B* and *Γ* are path coefficient matrices and *ζ* are error vectors. The basic assumptions are that the mean values of *δ* and *ε* are 0, the mean value of *ζ* is 0, there is no serial correlation between *δ* and *ε* or between *ζ* and *η*, and *ζ* is not relevant to *η*, *ε*, and *δ*.

### 4.2 Reliability and validity test

#### 4.2.1 Reliability analysis

In this study, SPSS 23.0 was used for reliability and validity testing. The results showed that the Cronbach’s α coefficient of the three scales was greater than 0.7, while the Cronbach’s α coefficient of the total scale was 0.824. Therefore, the questionnaire had good internal consistency reliability, reasonable question setting, and reliable data.

#### 4.2.2 Validity analysis

The KMO and Barlett’s sphericity test P-values of 11 items on social adaptability were calculated using sociological factor analysis. The results showed that the KMO value was 0.830 and Barlett’s sphericity test was significant (p<0.001), indicating that the data were correlated and that the correlation coefficient matrix of factors was not an identity matrix. It can extract the least number of factors while explaining most of the variance, which meets the research requirements and can be analyzed in the next step. The validity test of 21 variables related to relocation satisfaction shows that the common degree of all indicators is greater than 0.5, which meets the requirements of factor analysis and has high efficiency.

The principal component analysis method was used to extract the components of the observed variables reflecting relocation adaptability in the questionnaire, and three common factors were extracted by varimax rotation. It can be seen from Table 3 that the minimum eigenvalue of the factor is 1.892, which meets the requirements of factor analysis. The cumulative variance explained by these three factors is 65.770%, which can explain most of the variance. After testing the factor loading coefficients pointed by each observed variable, all of the factor loading coefficients are higher than 0.6. Therefore, in general, the indicator measurement of the social adaptability dimension of labor migrants is reliable. The Columbach alpha coefficient of the scale is large, and the reliability coefficient of the whole scale reaches 0.830, which indicates good construct validity in view of the high factor loading. Preliminary results show that the variable selection of social adaptability factors and the predictive items are appropriate.

**Table 3:** Factor analysis results of the labor migration relocation adaptability scale.

		Principal factor		
		1	2	3
Method of income		.880	--	--
Mode of employment		.854	--	--
Mode of consumption		.613	--	--
Part-time life		--	.746	--
Being discriminated against		--	.726	--
Status of immigrant		--	.717	--
With a native		--	--	.809
Language environment		--	--	.787
Mode of communication		--	--	.662
Before rotation	Value of characteristic	3.771	1.324	.824
	Percentage of explained variance (%)	41.902	14.715	9.153
	Cumulative percentage of explained variance (%)	41.902	56.617	65.770
After rotation	Value of characteristic	2.066	1.961	1.892
	Percentage of explained variance (%)	22.955	21.792	21.023
	Cumulative percentage of explained variance (%)	22.955	44.747	65.770

Extraction method: principal component analysis.

Rotation method: Caesar normalized maximum variance method.

a. The rotation has converged after six iterations.

#### 4.2.3 Model testing and optimization

As shown in Table 4, The initial structural equation model included 4 latent variables and 13 observable variables, Confirmatory factor analysis of the model was conducted to test whether economic, social and cultural adaptability can truly reflect the adaptability of migrants. The maximum likelihood method given by SPSS Amos 26.0 was used to estimate the parameters of the model, and finally the parameter estimation results of the model and the standardized path system were obtained. The critical ratio method was used to test the significance of the path coefficient (see Table 4). The results show that among the 16 observed variable and latent variable paths and 3 latent variable and latent variable paths, (1) "Employment stability <—cultural adaptability" fails the significance test; (2) "Employment stability <—social adaptability" passes the significance test at the level of 0.05; (3) The rest pass the significance test at the 0.01 level.

**Table 4:** Path (load) coefficient output result.

			Estimate	S.Estimate	S.E.	C.R.	P
Society	<---	Economy	0.757	0.687	0.094	8.086	***
Culture	<---	Economy	0.466	0.501	0.075	6.256	***
Stability of employment	<---	Society	0.229	0.194	0.109	2.094	0.036
Stability of employment	<---	Economy	0.813	0.624	0.129	6.323	***
Stability of employment	<---	Culture	0.064	0.046	0.096	0.670	0.503
Mode of consumption S	<---	Economy	1.000	0.552			
Mode of employment S	<---	Economy	1.476	0.860	0.142	10.375	***
Method of income S	<---	Economy	1.342	0.777	0.131	10.225	***
Mode of communication S	<---	Culture	1.000	0.703			
Language environment S	<---	Culture	0.974	0.638	0.108	9.052	***
With native S	<---	Culture	0.943	0.680	0.103	9.122	***
Immigration status S	<---	Society	1.000	0.715			
Being discriminated against S	<---	Society	0.888	0.619	0.094	9.456	***
Spare time in life S	<---	Society	0.976	0.671	0.094	10.350	***
Employment policy M	<---	Stability of employment	1.000	0.694			
Employment environment M	<---	Stability of employment	1.007	0.746	0.080	12.565	***
Member employment M	<---	Stability of employment	1.056	0.722	0.087	12.075	***
Self-assessment of employment P	<---	Stability of employment	0.965	0.622	0.092	10.532	***

* “***”“**”“*” indicates that the path coefficients are significant at the 1%, 5%, and 10% levels, respectively. The four paths with "-" represent the benchmark for SEM parameter estimation, which are taken as significant paths to estimate whether other paths are significant.

Because the research group personally felt that cultural adaptability had a strong role in promoting employment stability during the investigation, it revised the model, as shown in Table 5 below, and achieved good results.

**Table 5:** Model modification process.

Path of correction			Reasons for amendment
e7	<-->	e16	The adaptability of immigration status is based on a certain culture, and there is a close connection between the adaptability of labor immigration status (e7) and its own cultural adaptability (e16)
e1	<-->	e15	The adaptability of consumption patterns is based on certain environmental changes, and labor migrants must adapt to the consumption patterns in the place of destination
e9	<-->	e16	The adaptability of spare time life is based on a certain culture, and there is a close relationship between the adaptability of spare time in life of labor immigrants (e9) and their own cultural adaptability (e16)
e6	<-->	e8	The adaptability of the relationship with locals is closely related to the adaptability of being discriminated against. The stronger the adaptability of the relationship with locals (e6) is, the smaller the possibility of being discriminated against (e8) is, that is, the stronger the adaptability of being discriminated against is, and vice versa

As can be seen from Table 6, after the correction, the significance of the path is significantly enhanced, and the value of the path coefficient has also undergone some changes.

**Table 6:** Comparison of the results before and after the correction.

				Estimate	S.Estimate	S.E.	C.R.	P
Before correction	Stability of employment	<--	Society	0.229	0.194	0.109	2.094	0.036
	Stability of employment	<--	Economy	0.813	0.624	0.129	6.323	***
	Stability of employment	<--	Culture	0.064	0.046	0.096	0.670	0.503
After correction	Stability of employment	<--	Society	0.253	0.206	0.101	2.510	0.012
	Stability of employment	<--	Economy	0.785	0.567	0.126	6.226	***
	Stability of employment	<--	Culture	0.188	0.134	0.090	2.096	0.036

* “***”“**”“*” the path coefficients are significant at 1%, 5%, and 10%, respectively.

First, the significance of "employment stability <—culture" after modification is p = 0.036; that is, it is significant under p<0.05. The standardized path coefficient has also increased to 0.134, an increase of 0.88.

Second, after the modification, the significance of "employment stability <—society" has also increased from 0.036 to 0.012, and the path coefficient has increased from 0.194 to 0.206, an increase of 0.012.

Third, after the modification, the significance of "employment stability <- economy" remains unchanged, while the path coefficient has decreased by 0.057 from 0.624 to 0.567, but the overall impact is not significant.

### 4.3 Model fitting

For the structural equation model, we have summarized the fit index of the prevailing model, and the specific results are shown in Table 7. All of the indicators after modification meet the requirements, so the goodness of fit of the model is excellent and in line with expectations.

**Table 7:** Fitting degree of AMOS-modified model.

Model Fitting Index	Optimal Standard Value	Statistical Value	Fitting Condition
CMIN/DF	<3	1.933	good
GFI	>0.9	0.960	good
AGFI	>0.9	0.935	good
RMSEA	<0.08	0.049	good
NFI	>0.9	0.939	good
TLI	>0.9	0.957	good
CFI	>0.9	0.969	good
IFI	>0.9	0.970	good
PNFI	>0.5	0.674	good
PCFI	>0.5	0.696	good

### 4.4 Hypothesis structure test analysis

**Fig 2:**
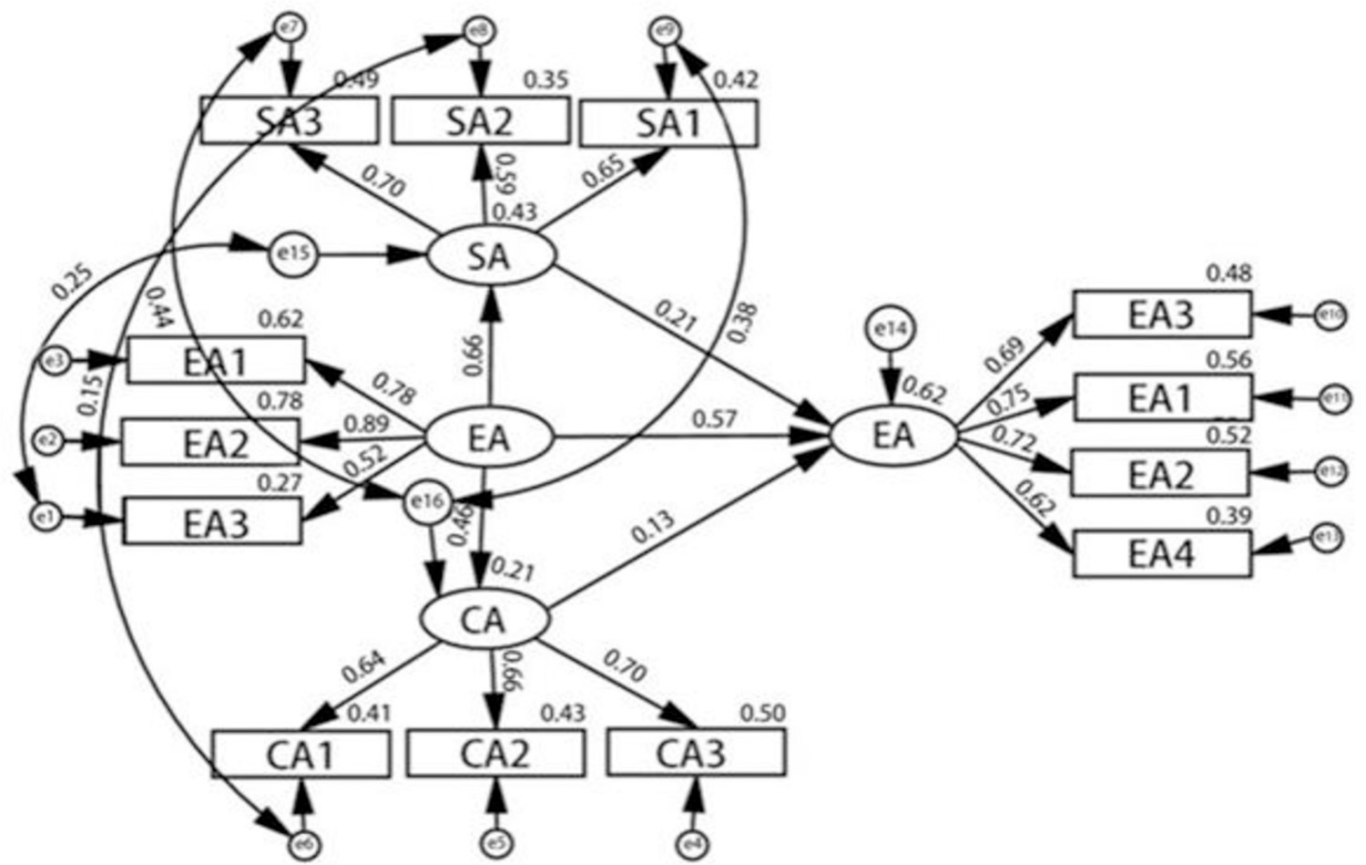
Structural equation of the relationship between the relocation adaptability and employment stability of labor migrants.

The optimized structural equation model diagram is shown in Fig 2. From Fig 2, “Structural equation diagram of the relationship between relocation adaptability of labor migrants and employment stability," the mutual relationship between various potential variables can be observed.

1. The relationship between exogenous and endogenous latent variables

First, the standardized path coefficient of economic adaptability on employment stability is 0.567, and it is significant at the level of 1% (p<0.01), which indicates that economic adaptability has a positive effect on employment stability. Accordingly, the original hypothesis 1, "There is a positive effect between economic adaptability and the employment stability of labor migrants," is valid.

Second, the standardized path coefficient of social adaptability on employment stability is 0.206, and it is significant at the level of 5% (p<0.05), which indicates that social adaptability has a positive effect on employment stability. Accordingly, the original hypothesis 2, "There is a positive effect between social adaptability and the employment stability of labor migrants," is valid.

Third, the standardized path coefficient of cultural adaptability on employment stability is 0.134, and it is significant at the level of 5% (p<0.05), which indicates that cultural adaptability has a positive effect on employment stability. Accordingly, the original hypothesis 3, "There is a positive effect between acculturation and the employment stability of labor migrants," is valid.

2. The relationship between exogenous latent variables and exogenous latent variables

First, among the three exogenous variables, economic adaptation has a decisive role.

Second, in terms of significance, economic adaptation is significant at P<0.01, as are social adaptation and cultural adaptation, and all of them have a positive effect.

Third, the value of the path coefficient shows that the effect of economic adaptation on social adaptation (0.655) is greater than that on cultural adaptation (0.459), which indicates that the change of social adaptation is stronger than the change of cultural adaptation in the case of increasing economic adaptation, and relatively speaking, the change of cultural adaptation is slower than the change of economic adaptation and social adaptation.

3.The relationship between observable variables and latent variables

First, as can be seen from the table, in regard to economic adaptability, the degrees of the direct influence of employment mode adaptability, income mode adaptability, and consumption mode adaptability are 0.886, 0.785, and 0.519, respectively, all of which are positive; the first two are more than 0.6, which indicates that their influence on economic adaptability is large. In particular, the coefficient of employment mode adaptability is as high as 0.886; consumption mode adaptability is lower, at 0.519, with a relatively small influence on economic adaptability.

Secondly as can be seen from the table, among the social adaptations, the degrees of the direct influence of immigration status adaptation, amateur life adaptation, and being discriminated against adaptation are 0.700, 0.647, and 0.588, respectively. All of them are positive, and the first two are more than 0.6, which indicates that their influence on social adaptations is large. In particular the immigration status adaptation coefficient is 0.700; the adaptation of being discriminated against is lower, at 0.588, meaning it has relatively less impact on social adaptability.

Third, as can be seen from the table, in the case of cultural adaptability, the degrees of the direct influence of adaptability of interaction mode, adaptability of language environment, and adaptability with natives are 0.704, 0.659, and 0.637, respectively. All of them are positive influences, and all are more than 0.6, which indicates that all aspects have a greater influence on cultural adaptability. In particular, the adaptability coefficient of interaction mode is 0.704.

Fourth, s can be seen from the above table, regarding employment stability, the degrees of direct influence of employment environment satisfaction, members’ employment satisfaction, employment policy satisfaction, and employment self-assessment are 0.748, 0.721, 0.695, and 0.623, respectively. All of them have a positive influence, and all are more than 0.6, which indicates that all aspects have a greater influence on employment stability. In particular, the coefficient of satisfaction with the employment environment is 0.748.

## Conclusions and discussion

### 5.1 Conclusions

Based on the structural equation of the relationship between labor migrant relocation satisfaction and employment stability in Fig 1 and the output of the path coefficient of the model, the effect of labor migrant relocation satisfaction on employment stability is calculated as shown in Table 8.

**Table 8:** Latent variable effect.

Latent variable		Dimensionality	Effect on employment stability		
Observed variable	Path coefficient		Total effect	Direct effect	Indirect effect
Consumption pattern	0.519	Economic adaptability	0.763	0.567	0.196
Employment mode	0.886				
Income pattern	0.785				
Immigration status	0.700	Social adaptability	0.206	0.206	--
Be discriminated against	0.588				
Spare time in life	0.647				
Way of communication	0.704	Cultural adaptation	0.134	0.134	--
Language environment	0.659				
With the locals	0.637				

First, based on the analysis of the economic adaptability dimension, the total effect of economic adaptability on employment stability is 0.763, which is the largest among the three; it has both a direct positive effect of 0.567 and an indirect effect of 0.196. It shows that higher economic adaptability can effectively improve the stability of the employment of labor migrants and effectively guarantee that the residents in the place of relocation will have a relatively stable job, thus promoting the realization of the goal of poverty alleviation and enrichment. From the perspective of the three observational variables of economic adaptability, the degrees of the direct influence of the adaptability of employment mode, the adaptability of income mode, and the adaptability of consumption mode are 0.886, 0.785, and 0.519, respectively, among which the adaptability of employment mode is the highest (0.886), which indicates that only when the adaptability of employment mode is continuously improved will the labor migrants gradually improve their adaptability of the income and consumption modes. There is a close connection between the three, and they promote and improve each other.

Second, based on the analysis of the dimension of social adaptability, the degree of the direct effect of social adaptability on employment stability is 0.206, which is relatively small. Among the three observed variables of social adaptability, the degrees of direct influence of immigrant status adaptability, leisure life adaptability and discrimination adaptability are 0.700, 0.647 and 0.588, respectively, among which immigrant status adaptability is the highest (0.700), indicating that only the adaptability of constantly improving immigrant status can facilitate integration into the local society. Only in this way can individuals gradually integrate into the local life and eradicate the discriminatory and heterogeneous influence of others on labor immigrants. The relationship among the three is extremely close, and only by constantly improving the adaptability of being discriminated against can individuals take the initiative to improve all aspects of social adaptability.

Third, based on the analysis of the cultural adaptability dimension, cultural adaptability has a positive direct effect of 0.134 on employment stability, which is the smallest among the three. This indicates that for every unit increase in the cultural adaptability of labor migrants to the area of residence, the local employment stability will increase by 0.134 units. Among the three observed variables of cultural adaptability, the degrees of direct influence of communication style adaptability, language environment adaptability, and local adaptability are 0.704, 0.659, and 0.637, respectively. The adaptability of communication mode, language environment, and local people all reflect the initiative of labor migrants. From the mountain area to the Sichuan area, or relocation within the county, the language environment of labor migrants and the adaptability of the way of communication are strong, with the same dialect and the same local conditions and customs. Relatively speaking, the relationship with the local people must exist through active contact to to better integrate into the local cultural life, including economic and non-economic aspects, such as finding a job, children’s school, and local marriage.

### 5.2 Discussion

About 5,000 urban communities formed during the 13th Five-Year Plan period are in the initial stage of development and face many urgent problems to be solved. According to the above research conclusions, this study will provide the following experience and theoretical guidance for other similar or similar regions in Ningxia, so that they can avoid detors and speed up the pace, and inject vitality into the comprehensive, coordinated and sustainable development of labor migrant communities.

1. Enhance economic adaptability, stabilize employment, and increase income.

Labor migration is an initiative organized effectively by the government, with the active participation of labor migrants in an attempt to free themselves from the constraints of the natural environment and to achieve the goal of poverty alleviation and enrichment, with stable employment and increased income as its core elements. Labor migrants must actively change their various economic behaviors to adapt to the new requirements of the new environment. First, they must establish a good concept of employment before choosing a job and abandon the idea of dependence and passivity. T It is especially important for young women to change the phenomenon that they can have a job but do not want to do it. They must go to poverty alleviation workshops and do some odd jobs that can be done at home. Only by adopting positive employment behaviors and achieving effective and stable employment can family incomes be increased. Second, labor migrants need to actively seek other sources of income. In addition to obtaining corresponding income through physical labor and relevant national policy subsidies, they should also consider revitalizing existing assets and increasing capital income, for example, through rational financial management. Finally, labor migrants must take the initiative to adapt to changing consumption patterns. This is most challenging, especially for long-distance migrants. Traditional consumption patterns cannot be sustained without the support of land-based output. To adapt to urban consumption patterns, it is necessary to engage in active and proactive employment in order to earn an income and make the transition from traditional consumption patterns based on subsistence production to those based on monetary exchange.

2. Enhance social adaptability, take the initiative to adapt, and participate actively.

Labor migration is a government-organized attempt to change the development environment for the poor and achieve sustainable development through relocation, with proactive adaptation and active participation as its core elements. Labor migrants must change their social behaviors to meet the new requirements of the new environment. First, they should take the initiative to adapt to and correctly treat their own migrant status. On one hand, the existence of the migrant status for their access to a large amount of social support provides convenience and is conducive to their own development but may also lead to holding the "wait and rely on the demand, lazy, loose, and slow" idea to provide a protective umbrella. On the other hand, the existence of migrant status will widen the distance between migrants and local residents, leading them to lose many opportunities to utilize social resources for their own development. Therefore, while actively seeking external support, labor migrants must also weaken the impact of immigration status on themselves and strive for more development opportunities. Second, they must take the initiative to adapt to and appropriately manage the discrimination they may encounter. On one hand, being discriminated against is the threshold effect that new members will inevitably encounter when they enter a new environment and is a common social phenomenon; on the other hand, the daily behaviors and language differences of labor migrants may also lead to misunderstandings and misinterpretations, which is a transient social phenomenon. Therefore, labor migrants need to take the initiative to regulate and constrain their behavior according to the requirements of the new environment, improve their overall quality, and reduce or eliminate the existence of this phenomenon. Finally, they must take the initiative to adapt to and appropriately manage the new changes and diversification of their spare time in life. Spare time can be divided into two categories: those that have a positive effect on the individual and those that may have a negative effect on the individual. The former can facilitate making friends, improving communication, obtaining information, and personal development, while the latter may cause people to become disorganized and depressed and even damage family harmony or violate the law and discipline. Therefore, labor migrants need to distinguish between the good and the bad in accordance with the changing situation and choose a way of life that suits them so that they can enrich and improve themselves.

3. Strengthen cultural adaptability, actively seek change, and actively adapt.

Labor migration is a form of migration activity that includes cultural change, including the change from closed culture to open culture and from rural culture to urban culture, with proactive change-seeking and active adaptation as its core content. Labor migrants must effectively change their own cultural behaviors to adapt to the new requirements of the new environment for their own development. First, it is necessary to continuously strengthen the adaptability of the language environment. Language is an important part of culture. Although labor migration is carried out within Ningxia, the language and even the culture are the same, but there are still some regional differences. This requires that labor migrants should take the initiative to learn the language and culture of the place they move to, adapt to the local language characteristics, and lay a good foundation for deepening and increasing communication. Second, it is necessary to continuously improve the adaptability of the mode of communication. The ways of communication are diverse and include the positive mode of mutual benefit and win-win situations as well as the negative mode of harming others and oneself or even losing for both sides. This requires that labor migrants should choose positive ways of interaction and, while seeking help and development, give more to others, thus obtaining more opportunities for development. Finally, the ability to communicate with locals should be continuously enhanced. Strengthening communication with people is an important method to quickly realize cultural change. Only through constant contact can one familiarize oneself with the culture of the place of migration, understand the connotation, characteristics, and differences of the culture, and then realize intercultural communication and adaptation. This requires that labor migrants should increase their exchanges with local people, gradually adapt to and integrate into the local culture, and lay a good cultural foundation for long-term development.

## Supporting information

S1 Data (XLSX)

S1 File (PDF)

S2 File (PDF)
