# Supplementary material for: Research on the influence of relocation adaptability on the employment stability of Chinese-style labor immigration: The case of labor migrant communities in Yinchuan City, Ningxia, China
Source: PLoS One. 2024 Jun 6;19(6):e0304199. doi: 10.1371/journal.pone.0304199 (PMC11156335; doi:10.1371/journal.pone.0304199)
Supplement: S1 File — (PDF) [file pone.0304199.s002.pdf]

Research on the influence of relocation adaptability on employment stability of Chinese-style labor immigration

Question 1 Region of location [Single choice]

options (as in computer software settings)	Subtotal	proportions
Zhanzheng township in Yunnan	162	39.04%
Wangyuan township, Hainan	141	33.98%
Minning Township, Taiwan	101	24.34%
Lingwu prefecture level city in Gansu	2	0.48%
Guyuan city and prefecture in Ningxia	4	0.96%
sea source	0	0%
Shizuishan prefecture level city in Ningxia on the border with Inner Mongolia	1	0.24%
(sth. or sb) else	4	0.96%
Number of valid entries for this question	415	

Question 2 A01 Your Gender [Single Choice]

options (as in computer software settings)	Subtotal	proportions
1. Male	208	50.12%
2. Women	207	49.88%
Number of valid entries for this question	415	

Question 3 A02 Your age ( ) years [fill in the blanks]

For fill-in-the-blank data, please download the detailed data.

Question 4 A03 Your marital status [Single Choice]

options (as in computer software settings)	Subtotal	proportions
1. Yes	370	89.16%
2. No	45	10.84%
Number of valid entries for this question	415	

Question 5 A04 You immigrated in ( ) years [fill in the blanks]

Fill-in-the-blank data can be obtained by downloading the detailed data.

Question 6 A05's family status prior to relocation is [Single Choice]

options (as in computer software settings)	Subtotal	proportions
1. Poor households with documented cards	219	52.77%
2. Non-filing poor households	69	16.63%
3. Households out of poverty	11	2.65%
4. General households	115	27.71%
5. Wealthy households	1	0.24%
Number of valid entries for this question	415	

Question 7 The status of the family after A06's relocation is [Single Choice].

options (as in computer software settings)	Subtotal	proportions
1. Poor households with documented cards	181	43.61%
2. Non-filing poor households	82	19.76%
3. Households out of poverty	32	7.71%

4. General households	119	28.67%
5. Wealthy households	1	0.24%
Number of valid entries for this question	415	

Question 8 A07 Nation [Single-Choice]

options (as in computer software settings)	Subtotal	proportions
1. Han Chinese	185	44.58%
2. Hui	230	55.42%
3. Other	0	0%
Number of valid entries for this question	415	

Question 9 A08 Your monthly salary $ [Single choice]

options (as in computer software settings)	Subtotal	proportions
Less than 1.2000	226	54.46%
2.2000-3000	117	28.19%
3.3000-4000	51	12.29%
4.4000-5000	14	3.37%
5.5000 or more	7	1.69%
Number of valid entries for this question	415	

Question 10 A09 Your family's total income for the year $10,000 [Single Choice]

options (as in computer software settings)	Subtotal	proportions
Below 1.2	148	35.66%
2.2-3	143	34.46%

3.3-4	76	18.31%
4.4-5	23	5.54%
5.5 or more	25	6.02%
Number of valid entries for this question	415	

Question 11 A10 There are ( ) people in your household [fill in the blanks]

For fill-in-the-blank data, please download the detailed data.

Question 12 A11 Your household has a labor force of ( ) [fill in the blank]

For fill-in-the-blank data, please download the detailed data.

Question 13 A12 There are old people and children ( ) in your household [fill in the blanks]

For fill-in-the-blank data, please download the detailed data.

Question 14 The area of your home is ( ) square meters [fill in the blanks].

For fill-in-the-blank data, please download the detailed data.

Question 15 B01 Your level of education [Single choice]

options (as in computer software settings)	Subtotal	proportions
1. Below elementary school	153	36.87%
2. Primary school	84	20.24%
3. Middle school	98	23.61%
4. High school or secondary school	45	10.84%
5. Tertiary	20	4.82%

6. Undergraduate	15	3.61%
Number of valid entries for this question	415	

Question 16 B02 Status of your participation in skills training [Single Choice]

options (as in computer software settings)	Subtotal	proportions
1. Participated in	123	29.64%
2. Non-participation	292	70.36%
Number of valid entries for this question	415	

Question 17 B03 Health Status [Single Choice]

options (as in computer software settings)	Subtotal	proportions
1. Health	315	75.9%
2. Other	100	24.1%
Number of valid entries for this question	415	

Question 18 C0101 Your overall satisfaction status with the relocation [Single Choice]

options (as in computer software settings)	Subtotal	proportions
1. Very satisfied	46	11.08%
2. More satisfied	184	44.34%
3. General	83	20%
4. Less satisfactory	77	18.55%
5. Very dissatisfied	25	6.02%

Number of valid entries for this question	415	

Question 19 C0201 How satisfied are you with your current level of household income [Single Choice]

options (as in computer software settings)	Subtotal	proportions
1. Very satisfied	13	3.13%
2. More satisfied	112	26.99%
3. General	108	26.02%
4. Less satisfactory	157	37.83%
5. Very dissatisfied	25	6.02%
Number of valid entries for this question	415	

Question 20 C0202 How satisfied are you with your current household living conditions [Single Choice]

options (as in computer software settings)	Subtotal	proportions
1. Very satisfied	32	7.71%
2. More satisfied	143	34.46%
3. General	111	26.75%
4. Less satisfactory	106	25.54%
5. Very dissatisfied	23	5.54%
Number of valid entries for this question	415	

Question 21 C0203 How satisfied are you with your current family harmony [Single Choice]

options (as in computer software settings)	Subtotal	proportions
1. Very satisfied	127	30.6%
2. More satisfied	223	53.73%
3. General	52	12.53%
4. Less satisfactory	11	2.65%
5. Very dissatisfied	2	0.48%
Number of valid entries for this question	415	

Question 22 C0204 How satisfied are you with the quality of life of your family now [Single Choice]

options (as in computer software settings)	Subtotal	proportions
1. Very satisfied	37	8.92%
2. More satisfied	175	42.17%
3. General	131	31.57%
4. Less satisfactory	66	15.9%
5. Very dissatisfied	6	1.45%
Number of valid entries for this question	415	

Question 23 C0205 How satisfied are you with the current employment status of your family members [Single Choice]

options (as in computer software settings)	Subtotal	proportions
1. Very satisfied	25	6.02%
2. More satisfied	128	30.84%
3. General	117	28.19%

4. Less satisfactory	129	31.08%
5. Very dissatisfied	16	3.86%
Number of valid entries for this question	415	

Question 24 C0301 How satisfied are you with the current community policing environment [Single Choice]

options (as in computer software settings)	Subtotal	proportions
1. Very satisfied	59	14.22%
2. More satisfied	230	55.42%
3. General	80	19.28%
4. Less satisfactory	36	8.67%
5. Very dissatisfied	10	2.41%
Number of valid entries for this question	415	

Question 25 C0302 How satisfied are you with the current community relations among the officers [Single Choice]

options (as in computer software settings)	Subtotal	proportions
1. Very satisfied	59	14.22%
2. More satisfied	201	48.43%
3. General	108	26.02%
4. Less satisfactory	43	10.36%
5. Very dissatisfied	4	0.96%
Number of valid entries for this question	415	

Question 26 C0303 How satisfied are you with your current community health environment [Single Choice]

options (as in computer software settings)	Subtotal	proportions
1. Very satisfied	71	17.11%
2. More satisfied	249	60%
3. General	63	15.18%
4. Less satisfactory	24	5.78%
5. Very dissatisfied	8	1.93%
Number of valid entries for this question	415	

Question 27 C0304 How satisfied are you with your current neighborhood in your community [Single Choice]

options (as in computer software settings)	Subtotal	proportions
1. Very satisfied	80	19.28%
2. More satisfied	254	61.2%
3. General	71	17.11%
4. Less satisfactory	9	2.17%
5. Very dissatisfied	1	0.24%
Number of valid entries for this question	415	

Question 28 C0305 How satisfied are you with the current management of your community [Single Choice]

options (as in computer software settings)	Subtotal	proportions

1. Very satisfied	44	10.6%
2. More satisfied	225	54.22%
3. General	105	25.3%
4. Less satisfactory	35	8.43%
5. Very dissatisfied	6	1.45%
Number of valid entries for this question	415	

Question 29 C0401 Your Satisfaction with Labor Migrant Housing Policies [Single-Choice Question]

options (as in computer software settings)	Subtotal	proportions
1. Very satisfied	46	11.08%
2. More satisfied	173	41.69%
3. General	93	22.41%
4. Less satisfactory	80	19.28%
5. Very dissatisfied	23	5.54%
Number of valid entries for this question	415	

Question 30 C0402 Your Satisfaction with the Employment Policies for Labor Migrants [Single Choice]

options (as in computer software settings)	Subtotal	proportions
1. Very satisfied	35	8.43%
2. More satisfied	140	33.73%
3. General	135	32.53%
4. Less satisfactory	87	20.96%

5. Very dissatisfied	18	4.34%
Number of valid entries for this question	415	

Question 31 C0403 Your Satisfaction Status with Labor Migrant Health Care Policy [Single Choice]

options (as in computer software settings)	Subtotal	proportions
1. Very satisfied	54	13.01%
2. More satisfied	199	47.95%
3. General	105	25.3%
4. Less satisfactory	48	11.57%
5. Very dissatisfied	9	2.17%
Number of valid entries for this question	415	

Question 32 C0404 Your Satisfaction with the Pension Policy for Labor Migrants [Single-Choice Question]

options (as in computer software settings)	Subtotal	proportions
1. Very satisfied	40	9.64%
2. More satisfied	182	43.86%
3. General	141	33.98%
4. Less satisfactory	44	10.6%
5. Very dissatisfied	8	1.93%
Number of valid entries for this question	415	

Question 33 C0405 Your satisfaction status with the low income policy for labor

migrants [Single Choice]

options (as in computer software settings)	Subtotal	proportions
1. Very satisfied	38	9.16%
2. More satisfied	146	35.18%
3. General	144	34.7%
4. Less satisfactory	67	16.14%
5. Very dissatisfied	20	4.82%
Number of valid entries for this question	415	

Question 34 C0406 Your Satisfaction with the Financial Policies for Labor Migration [Single-Choice Question]

options (as in computer software settings)	Subtotal	proportions
1. Very satisfied	24	5.78%
2. More satisfied	122	29.4%
3. General	212	51.08%
4. Less satisfactory	48	11.57%
5. Very dissatisfied	9	2.17%
Number of valid entries for this question	415	

Question 35 C0501 How satisfied are you with your local transportation environment [Single Choice]

options (as in computer software settings)	Subtotal	proportions
1. Very satisfied	82	19.76%

2. More satisfied	254	61.2%
3. General	58	13.98%
4. Less satisfactory	15	3.61%
5. Very dissatisfied	6	1.45%
Number of valid entries for this question	415	

Question 36 C0502 How satisfied are you with your local educational environment [Single Choice]

options (as in computer software settings)	Subtotal	proportions
1. Very satisfied	54	13.01%
2. More satisfied	248	59.76%
3. General	90	21.69%
4. Less satisfactory	23	5.54%
5. Very dissatisfied	0	0%
Number of valid entries for this question	415	

Question 37 C0503 How satisfied are you with your local health care environment [Single Choice]

options (as in computer software settings)	Subtotal	proportions
1. Very satisfied	46	11.08%
2. More satisfied	230	55.42%
3. General	107	25.78%
4. Less satisfactory	28	6.75%
5. Very dissatisfied	4	0.96%

Number of valid entries for this question	415	

Question 38 C0504 Your satisfaction with your local ecological environment [Single Choice]

options (as in computer software settings)	Subtotal	proportions
1. Very satisfied	59	14.22%
2. More satisfied	266	64.1%
3. General	78	18.8%
4. Less satisfactory	11	2.65%
5. Very dissatisfied	1	0.24%
Number of valid entries for this question	415	

Question 39 C0505 How satisfied are you with the local employment environment [Single Choice]

options (as in computer software settings)	Subtotal	proportions
1. Very satisfied	29	6.99%
2. More satisfied	157	37.83%
3. General	132	31.81%
4. Less satisfactory	90	21.69%
5. Very dissatisfied	7	1.69%
Number of valid entries for this question	415	

Question 40 D01 How do you feel about your current identity status [Single Choice]

options (as in computer software settings)	Subtotal	proportions
1. Urbanites	165	39.76%
2. Others	250	60.24%
Number of valid entries for this question	415	

Question 41 D02 Status of your willingness to settle in the local area [Single Choice]

options (as in computer software settings)	Subtotal	proportions
1. Long-term	303	73.01%
2. Other	112	26.99%
Number of valid entries for this question	415	

Question 42 D03 Your relationship with local people is [Single Choice]

options (as in computer software settings)	Subtotal	proportions
1. Very cordial	78	18.8%
2. More cordial	259	62.41%
3. General	68	16.39%
4. Less cordial	9	2.17%
5. Very bad rapport	1	0.24%
Number of valid entries for this question	415	

Question 43 D04 How well you are adapting to your current employment style [Single Choice]

options (as in computer software settings)	Subtotal	proportions
1. Very adaptable	30	7.23%
2. Comparative adaptation	177	42.65%
3. General	134	32.29%
4. Less adaptable	65	15.66%
5. Very uncomfortable	9	2.17%
Number of valid entries for this question	415	

Question 44 D05 How well you are adapting to the way you are earning your income now [Single Choice]

options (as in computer software settings)	Subtotal	proportions
1. Very adaptable	16	3.86%
2. Comparative adaptation	174	41.93%
3. General	141	33.98%
4. Less adaptable	67	16.14%
5. Very uncomfortable	17	4.1%
Number of valid entries for this question	415	

Question 45 D06 How well you are adapting to the way you consume now [Single Choice]

options (as in computer software settings)	Subtotal	proportions
1. Very adaptable	26	6.27%
2. Comparative adaptation	146	35.18%
3. General	125	30.12%

4. Less adaptable	109	26.27%
5. Very uncomfortable	9	2.17%
Number of valid entries for this question	415	

Question 46 D07 How well you are adapting to your current housing style [Single Choice]

options (as in computer software settings)	Subtotal	proportions
1. Very adaptable	32	7.71%
2. Comparative adaptation	182	43.86%
3. General	103	24.82%
4. Less adaptable	84	20.24%
5. Very uncomfortable	14	3.37%
Number of valid entries for this question	415	

Question 47 D08 How well are you adapting to your current language environment [Single Choice]

options (as in computer software settings)	Subtotal	proportions
1. Very adaptable	93	22.41%
2. Comparative adaptation	239	57.59%
3. General	62	14.94%
4. Less adaptable	19	4.58%
5. Very uncomfortable	2	0.48%
Number of valid entries for this question	415	

Question 48 D09 How well are you adapting to the current way of social interaction [Single Choice]

options (as in computer software settings)	Subtotal	proportions
1. Very adaptable	46	11.08%
2. Comparative adaptation	268	64.58%
3. General	79	19.04%
4. Less adaptable	19	4.58%
5. Very uncomfortable	3	0.72%
Number of valid entries for this question	415	

Question 49 D10 How do you feel about the current situation of some people who do not look favorably on the adaptation of the labor migration situation [Single Choice]

options (as in computer software settings)	Subtotal	proportions
1. Very adaptable	24	5.78%
2. Comparative adaptation	167	40.24%
3. General	163	39.28%
4. Less adaptable	53	12.77%
5. Very uncomfortable	8	1.93%
Number of valid entries for this question	415	

Q50 D11 How well you are adapting to your current spare time lifestyle [Single Choice]

options (as in computer software settings)	Subtotal	proportions
1. Very adaptable	24	5.78%
2. Comparative adaptation	210	50.6%
3. General	125	30.12%
4. Less adaptable	46	11.08%
5. Very uncomfortable	10	2.41%
Number of valid entries for this question	415	

Question 51 D12 How well you have adapted to your status as a labor migrant

[Single Choice]

options (as in computer software settings)	Subtotal	proportions
1. Very adaptable	28	6.75%
2. Comparative adaptation	236	56.87%
3. General	108	26.02%
4. Less adaptable	34	8.19%
5. Very uncomfortable	9	2.17%
Number of valid entries for this question	415	

Question 52 D13 How well you are adapting to the present climatic conditions

[Single Choice]

options (as in computer software settings)	Subtotal	proportions
1. Very adaptable	61	14.7%
2. Comparative adaptation	255	61.45%
3. General	63	15.18%

4. Less adaptable	32	7.71%
5. Very uncomfortable	4	0.96%
Number of valid entries for this question	415	

Q53 D14 How well you are adapting to the way your community is managed now [Single Choice]

options (as in computer software settings)	Subtotal	proportions
1. Very adaptable	41	9.88%
2. Comparative adaptation	239	57.59%
3. General	87	20.96%
4. Less adaptable	38	9.16%
5. Very uncomfortable	10	2.41%
Number of valid entries for this question	415	

Question 54 E01 How would you rate your current employment situation [Single Choice]

options (as in computer software settings)	Subtotal	proportions
1. Very stable	18	4.34%
2. More stable	115	27.71%
3. General	109	26.27%
4. Less stable	131	31.57%
5. Very unstable	42	10.12%
Number of valid entries for this question	415	

Question 55 E02 Pathway status of your first job after relocating [Single Choice]

options (as in computer software settings)	Subtotal	proportions
1. Government-provided	77	18.55%
2. Provided by the government but not satisfied with the search on their own	27	6.51%
3. Find it yourself	257	61.93%
4. Other	54	13.01%
Number of valid entries for this question	415	

Question 56 E03 The status of the pathway you are currently working on [Single Choice]

options (as in computer software settings)	Subtotal	proportions
1. Government-provided	76	18.31%
2. Provided by the government but not satisfied with the search on their own	25	6.02%
3. Find it yourself	254	61.2%
4. Other	60	14.46%
Number of valid entries for this question	415	

Question 57 E04 Regional status of your employment [Single choice]

options (as in computer software settings)	Subtotal	proportions
1. Local	331	79.76%
2. Other	84	20.24%
Number of valid entries for this question	415	

Question 58 E05 Your current employment industry status [Single Choice]

options (as in computer software settings)	Subtotal	proportions
1. Accommodation and catering	32	7.71%
2. Wholesale and retail trade	13	3.13%
3. Construction	70	16.87%
4. Manufacturing	54	13.01%
5. Transportation	12	2.89%
6. Other	234	56.39%
Number of valid entries for this question	415	

Question 59 E06 Ownership status of the organization in which you are currently employed [Single Choice]

options (as in computer software settings)	Subtotal	proportions
1. State-owned enterprises	28	6.75%
2. Collective enterprises	11	2.65%
3. Private enterprise	161	38.8%
4. Non-governmental organizations	14	3.37%
5. Other	201	48.43%
Number of valid entries for this question	415	

Question 60 E07 Your daily working hours are ( ) hours [fill in the blanks]

For fill-in-the-blank data, please download the detailed data.

Question 61 E08 You work ( ) days per month [fill in the blanks]

For fill-in-the-blank data, please download the detailed data.

Question 62 E09 You work ( ) months per year [fill in the blanks]

For fill-in-the-blank data, please download the detailed data.

Question 63 E10 Status of your labor contract at the time of employment [Single choice]

options (as in computer software settings)	Subtotal	proportions
1. Signed	142	34.22%
2. Unsigned	273	65.78%
Number of valid entries for this question	415	

Question 64 E11 Status of your participation in just-sign training after employment [Single Choice]

options (as in computer software settings)	Subtotal	proportions
1. Trained	129	31.08%
2. No training	286	68.92%
Number of valid entries for this question	415	

Question 65 E12 Your city health insurance and worker's compensation status at the time of employment [Single Choice]

options (as in computer software settings)	Subtotal	proportions

1. Yes	155	37.35%
2. No	260	62.65%
Number of valid entries for this question	415	

Q66 E13 What do you think are the main reasons for employment stability (multiple choice, minimum three) [Multiple choice]

options (as in computer software settings)	Subtotal	proportions
1. Wage level	324	78.07%
2. Availability of social insurance	127	30.6%
3. Whether or not a contract has been signed	114	27.47%
4. Good and bad working environment	132	31.81%
5. Laxity in system management	35	8.43%
6. Personal dedication or lack thereof	29	6.99%
7. Individual skill level	103	24.82%
8. Distance from home	150	36.14%
9. Convenient transportation	93	22.41%
10. Heavy family burden	196	47.23%
11. Timeliness of payroll	138	33.25%
12. No	31	7.47%
Number of valid entries for this question	415	

Question 67 F01 Your attitude if you were allowed to go to school [Single Choice]

options (as in computer software settings)	Subtotal	proportions

1. Very willing	143	34.46%
2. More willing	143	34.46%
3. General	62	14.94%
4. Less willing	46	11.08%
5. Very reluctant	21	5.06%
Number of valid entries for this question	415	

Question 68 F02 If you were given the choice to go to school you would go to [Single Choice].

options (as in computer software settings)	Subtotal	proportions
1. Not on	72	17.35%
2. Primary school	28	6.75%
3. Middle School	35	8.43%
4. High School	64	15.42%
5. University	216	52.05%
Number of valid entries for this question	415	

Question 69 F03 How do you rate your self-worth [Single Choice]

options (as in computer software settings)	Subtotal	proportions
1. Very high	25	6.02%
2. Comparatively high	135	32.53%
3. General	210	50.6%
4. Comparatively low	40	9.64%
5. Very low	5	1.2%

Number of valid entries for this question	415	

Question 70 F04 How would you rate your own strengths [Single Choice]

options (as in computer software settings)	Subtotal	proportions
1. A lot	23	5.54%
2. More	151	36.39%
3. General	177	42.65%
4. Fewer	53	12.77%
5. Rarely	11	2.65%
Number of valid entries for this question	415	

Question 71 F05 How would you rate your self-confidence [Single Choice]

options (as in computer software settings)	Subtotal	proportions
1. Very strong	45	10.84%
2. Stronger	173	41.69%
3. General	147	35.42%
4. Weaker	45	10.84%
5. Very weak	5	1.2%
Number of valid entries for this question	415	

Question 72 F06 How would you rate your self-confidence [Single Choice]

options (as in computer software settings)	Subtotal	proportions
1. Very strong	42	10.12%
2. Stronger	177	42.65%

3. General	147	35.42%
4. Weaker	42	10.12%
5. Very weak	7	1.69%
Number of valid entries for this question	415	

Question 73 F07 How would you rate your own work ethic [Single Choice]

options (as in computer software settings)	Subtotal	proportions
1. Very diligent	114	27.47%
2. More diligent	205	49.4%
3. General	88	21.2%
4. Not very diligent	7	1.69%
5. Very un-industrious	1	0.24%
Number of valid entries for this question	415	

Question 74 F08 How would you rate your attitude towards accepting challenging work [Single Choice]

options (as in computer software settings)	Subtotal	proportions
1. Very willing	70	16.87%
2. More willing	199	47.95%
3. General	115	27.71%
4. Less willing	25	6.02%
5. Very reluctant	6	1.45%
Number of valid entries for this question	415	

Question 75 F09 How would you rate your socialization skills [Single Choice]

options (as in computer software settings)	Subtotal	proportions
1. Very strong	49	11.81%
2. Stronger	197	47.47%
3. General	135	32.53%
4. Weaker	28	6.75%
5. Very weak	6	1.45%
Number of valid entries for this question	415	

Question 76 F10 How would you rate your ability to achieve success [Single Choice]

options (as in computer software settings)	Subtotal	proportions
1. Very strong	39	9.4%
2. Stronger	164	39.52%
3. General	173	41.69%
4. Weaker	30	7.23%
5. Very weak	9	2.17%
Number of valid entries for this question	415	

Question 77 F11 How would you rate your intellectual curiosity [Single Choice]

options (as in computer software settings)	Subtotal	proportions
1. Very strong	61	14.7%
2. Stronger	158	38.07%
3. General	164	39.52%

4. Weaker	26	6.27%
5. Very weak	6	1.45%
Number of valid entries for this question	415	

Question 78 F12 How would you rate your own initiative in your work [Single Choice]

options (as in computer software settings)	Subtotal	proportions
1. Very active	78	18.8%
2. More active	217	52.29%
3. General	101	24.34%
4. Less active	17	4.1%
5. Very inactive	2	0.48%
Number of valid entries for this question	415	

Question 79 F13 How would you rate your own planning in your work [Single choice]

options (as in computer software settings)	Subtotal	proportions
1. Very strong	38	9.16%
2. Stronger	178	42.89%
3. General	158	38.07%
4. Weaker	30	7.23%
5. Very weak	11	2.65%
Number of valid entries for this question	415	

Question 80 F14 How would you rate your ability to overcome difficulties at work

[Single Choice]

options (as in computer software settings)	Subtotal	proportions
1. Very strong	49	11.81%
2. Stronger	179	43.13%
3. General	156	37.59%
4. Weaker	24	5.78%
5. Very weak	7	1.69%
Number of valid entries for this question	415	

Question 81 F15 How would you rate your ability to learn new things on the job

[Single Choice]

options (as in computer software settings)	Subtotal	proportions
1. Very strong	44	10.6%
2. Stronger	167	40.24%
3. General	150	36.14%
4. Weaker	39	9.4%
5. Very weak	15	3.61%
Number of valid entries for this question	415	

Q82 F16 How would you rate your ability to make a difference [Single Choice]

options (as in computer software settings)	Subtotal	proportions
1. Very strong	32	7.71%

2. Stronger	145	34.94%
3. General	161	38.8%
4. Weaker	59	14.22%
5. Very weak	18	4.34%
Number of valid entries for this question	415	

Question 83 F17 How would you rate your ability to grasp and apply policy [Single Choice]

options (as in computer software settings)	Subtotal	proportions
1. Very strong	21	5.06%
2. Stronger	124	29.88%
3. General	188	45.3%
4. Weaker	68	16.39%
5. Very weak	14	3.37%
Number of valid entries for this question	415	

Question 84 G01 What do you know most about labor migration policies (Multiple choice, minimum three) [Multiple choice]

options (as in computer software settings)	Subtotal	proportions
1. Housing	354	85.3%
2. Employment	239	57.59%
3. Training	58	13.98%
4. Education	182	43.86%
5. Medical insurance	231	55.66%

6. Old age	151	36.39%
7. Low-income insurance	152	36.63%
8. Finance	35	8.43%
9. Other	64	15.42%
Number of valid entries for this question	415	

Question 85 G02 What do you think is the most helpful of the labor migration policies (Multiple choice, minimum three) [Multiple choice]

options (as in computer software settings)	Subtotal	proportions
1. Housing	312	75.18%
2. Employment	188	45.3%
3. Training	42	10.12%
4. Education	207	49.88%
5. Medical insurance	231	55.66%
6. Old age	147	35.42%
7. Low-income insurance	127	30.6%
8. Finance	39	9.4%
9. Other	92	22.17%
Number of valid entries for this question	415	

Question 86 G03 The labor migration policies that you are most concerned about at the moment are (Multiple choice, minimum three) [Multiple choice]

options (as in computer software settings)	Subtotal	proportions
1. Housing	292	70.36%

2. Employment	292	70.36%
3. Training	63	15.18%
4. Education	216	52.05%
5. Medical insurance	208	50.12%
6. Old age	175	42.17%
7. Low-income insurance	153	36.87%
8. Finance	54	13.01%
9. Other	42	10.12%
Number of valid entries for this question	415	

Question 87 G04 The labor migration policies you would most like to see improved and refined are (Multiple choice, minimum three) [Multiple choice]

options (as in computer software settings)	Subtotal	proportions
1. Housing	299	72.05%
2. Employment	286	68.92%
3. Training	56	13.49%
4. Education	200	48.19%
5. Medical insurance	205	49.4%
6. Old age	195	46.99%
7. Low-income insurance	169	40.72%
8. Finance	60	14.46%
9. Other	60	14.46%
Number of valid entries for this question	415	
